# Time-course Transcriptome of *Parageobacillus thermoglucosidasius* DSM 6285 Grown in the Presence of Carbon Monoxide and Air

**DOI:** 10.3390/ijms21113870

**Published:** 2020-05-29

**Authors:** Habibu Aliyu, Teresa Mohr, Don Cowan, Pieter de Maayer, Anke Neumann

**Affiliations:** 1 Institute of Process engineering in Life Science 2: Technical Biology, Karlsruhe Institute of Technology, 76131 Karlsruhe, Germany; habibu.aliyu@partner.kit.edu (H.A.); mohr.teresa@gmail.com (T.M.); 2 Centre for Microbial Ecology and Genomics, Department of Biochemistry, Genetics and Microbiology, University of Pretoria, 0028 Pretoria, South Africa; don.cowan@up.ac.za; 3 School of Molecular & Cell Biology, Faculty of Science, University of the Witwatersrand, 2000 Johannesburg, South Africa; pieter.demaayer@wits.ac.za

**Keywords:** Carbon monoxide, hydrogen, *Parageobacillus thermoglucosidasius*, time-course, transcriptomics, and Water-Gas-Shift reaction

## Abstract

*Parageobacillus thermoglucosidasius* is a metabolically versatile, facultatively anaerobic thermophile belonging to the family *Bacillaceae*. Previous studies have shown that this bacterium harbours co-localised genes coding for a carbon monoxide (CO) dehydrogenase (CODH) and Ni-Fe hydrogenase (Phc) complex and oxidises CO and produces hydrogen (H_2_) gas via the water-gas shift (WGS) reaction. To elucidate the genetic events culminating in the WGS reaction, *P. thermoglucosidasius* DSM 6285 was cultivated under an initial gas atmosphere of 50% CO and 50% air and total RNA was extracted at ~8 (aerobic phase), 20 (anaerobic phase), 27 and 44 (early and late hydrogenogenic phases) hours post inoculation. The rRNA-depleted fraction was sequenced using Illumina NextSeq, v2.5, 1x75bp chemistry. Differential expression revealed that at 8 vs.. 20, 20 vs.. 27 and 27 vs.. 44 h post inoculation, 2190, 2118 and 231 transcripts were differentially (FDR < 0.05) expressed. Cluster analysis revealed 26 distinct gene expression trajectories across the four time points. Of these, two similar clusters, showing overexpression at 20 relative to 8 h and depletion at 27 and 44 h, harboured the CODH and Phc transcripts, suggesting possible regulation by O_2._ The transition between aerobic respiration and anaerobic growth was marked by initial metabolic deterioration, as reflected by up-regulation of transcripts linked to sporulation and down-regulation of transcripts linked to flagellar assembly and metabolism. However, the transcriptome and growth profiles revealed the reversal of this trend during the hydrogenogenic phase.

## 1. Introduction

Hydrogen gas (H_2_) has the highest energy per unit mass of all fuels (heating value of 141.9 MJ/kg) [1]. In contrast to traditional fuels, H_2_ is a zero-emission fuel when undergoing complete combustion in the presence of oxygen (2 H_2_ + O_2_ → 2 H_2_O) [2]. As such H_2_ has become prominent as a clean and sustainable alternative energy source [3]. Unlike industrial strategies for H_2_ production, such as coal gasification, steam reformation of natural gas and partial oxidation of oil, which require high energy inputs and rely on the use of non-renewable substrates [4], biohydrogen production is less energy intensive and the process results in almost zero waste products [4,5]. Biohydrogen is principally generated via biophotolysis of water by algae, and bacterial photo-fermentation and dark fermentation of organic substances [6]. However, these methods are constrained by both economic and technological limitations, including high costs of production and low yields [5]. Recently, there has been interest in biological production of H_2_ by microorganisms that couple hydrogenogenesis with the oxidation of carbon monoxide (CO) in the Water Gas Shift (WGS) reaction. A broad range of taxa including mesophilic anaerobes such as *Rhodospirillum rubrum* and *Rhodopseudomonas palustris* [7], thermophilic anaerobes such as *Carboxydothermus hydrogenoformans*, C. *pertinax* and *Thermosinus carboxydivorans* [8,9], and thermophilic archaea such as *Thermococcus onnurineus* [10] have been shown to tolerate high concentration of CO and oxidise this substrate via the WGS reaction [8,11]. 

The genus *Parageobacillus* comprises facultative anaerobic, obligate thermophilic (T^opt^ 55–65 °C) and catabolically diverse bacilli [12,13]. Because of the intrinsic capability to survive as spores, members of this genus have been isolated from a wide range of environments, ranging from extremely hot to temperate environments [14]. *Parageobacillus* spp. are of biotechnological interest as sources of thermostable enzymes including amylases, lipases, proteases and xylanases [15,16,17,18,19] and as whole-cell biocatalysts for biofuel production [20,21,22] and bioremediation [23]. Recently, *P. thermoglucosidasius* was demonstrated to produce H_2_ via the WGS reaction: CO + H_2_O → CO_2_ + H_2_ [24]. A carbon monoxide dehydrogenase (CODH) oxidises carbon monoxide (CO) and the resultant electrons are used by a hydrogen-evolving hydrogenase for the reduction of protons, resulting in the production of H_2_ and energy. This feature has been linked to a genetic locus harbouring three CODH genes (*cooCSF*) and 12 distinct genes (*phcA-L*) that constitute the Ni-Fe group 4a hydrogenase (Phc) [24,25,26]. 

*P. thermoglucosidasius* represents the first facultative anaerobe in which the WGS reaction has been observed. The organism grows aerobically until O_2_ is depleted, where after there is a lag phase during which biomass decreases. If CO is supplied, H_2_ is produced with a concomitant increase in biomass [27]. 

Here, to better understand the gene expression dynamics during the aerobic, lag and hydrogenogenic phases, RNA-seq was performed to elucidate the pattern of gene expression in *P. thermoglucosidasius* DSM 6285 grown with an initial gas atmosphere of 50% CO and 50% air.

## 2. Results

### 2.1. Differential Gene Expression during Aerobic, Anaerobic and Hydrogenogenic Growth Phases in P. thermoglucosidasius

**Figure 1:**
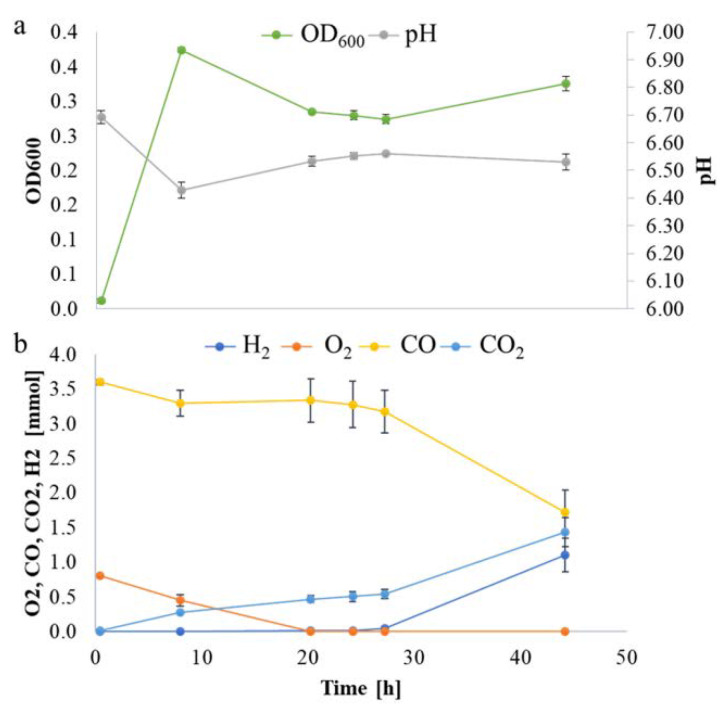
*P. thermoglucosidasius* DSM 6285 grown with an initial gas atmosphere composition of 50% CO and 50% air. (**a**) Absorbance (OD_600_) and pH measurements. (**b**) Gas composition.

*P. thermoglucosidasius* DSM 6285 was cultivated with an initial gas atmosphere of 50% air and 50% CO. Total RNA was extracted from cells harvested at four time points: 8, 20, 27, and 44 h post-inoculation. These timepoints approximately coincide with the middle of the aerobic growth phase, the early anaerobic phase when O_2_ is exhausted, when H_2_ is first detected and in the middle of the WGS phase, respectively (Figure 1). A total of ~106 million reads (7.9 Gb) were generated using Illumina NextSeq, v2.5, 1x75bp chemistry. The average number of reads ranged from ~1.46 × 10^7^ (mid-aerobic phase) to ~7.4 × 10^6^ (mid-WGS). Between 93% and 98% of these reads were trimmed and aligned to 3888 protein coding regions in the draft genome of *P. thermoglucosidasius* DSM 6285 (Table S1). A multidimensional scaling (MDS) plot, used to assess pattern of gene expression across the four sampling points revealed a clear separation of transcripts between the samples and commonality of expression between biological replicates within the sample groups (Figure S1). These results suggest that the transcriptome datasets are sufficiently robust to validly assess transcript dynamics across the studied conditions.

**Figure 2:**
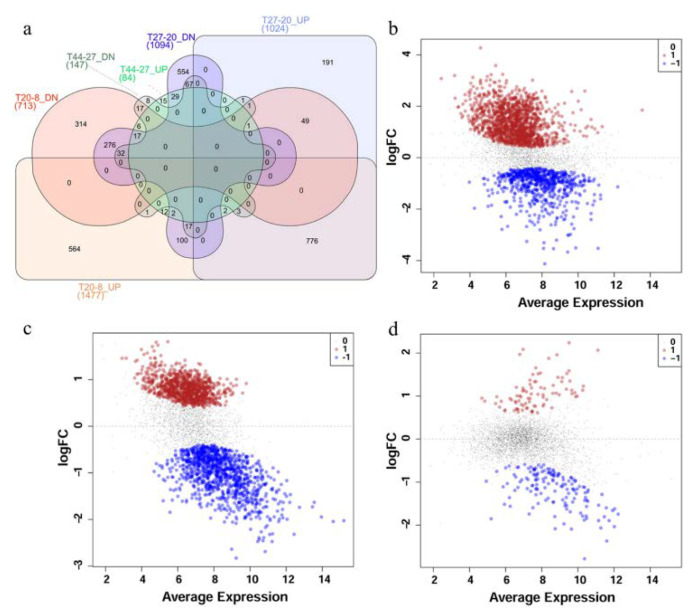
Differential expression of transcripts in *P. thermoglucosidasius* DSM 6285 grown with an initial gas atmosphere composition of 50% CO and 50% air. (**a**) Venn diagram showing the number of up- and down-regulated (UP and DN) transcripts for time points 20 vs.. 8 h (T20-8), 27 vs.. 20 h (T27-20) and 44 vs.. 27 h (T44-27). (**b**) MDS plot showing differential expression of transcripts at 20 vs.. 8 h post-inoculation, (**c**) MDS plot showing differential expression of transcripts at 27 vs.. 20 h post-inoculation and (**d**) MDS plot showing differential expression of transcripts t 44 vs.. 27 h post-inoculation. Red, black and blue dots represent upregulated, non-differentially expressed and down-regulated transcripts, respectively.

Comparisons of the expression profiles at the different time points revealed substantial differences between different phases. Comparison of the transcriptome profiles of *P. thermoglucosidasius* DSM 6285 at 8 and 20 h post-inoculation showed statistically significant (FDR < 0.05) differential expression of 2190 transcripts, of which ~67% and 33% were up- and down-regulated at 20 h, respectively (Figure 2a,b). In comparison to 20 h post-inoculation, 1024 and 1094 transcripts were significantly (FDR < 0.05) up- and down-regulated at 27 h, respectively (Figure 2a,c). By contrast, only 231 transcripts showed significant differential expression (FDR < 0.05) between the early (27 h) and late (44 h) WGS phases. Relative to 27 h, 84 and 147 transcripts were up- and down-regulated, respectively, at 44 h (Figure 2a,d). 

### 2.2. Metabolic and Functional Shifts in Response to Changing Gas Atmosphere

To determine the functional implication of the observed differences in expression profiles during the different growth phases, gene ontology (GO) and KEGG orthologues of the proteome of *P. thermoglucosidasius* DSM 6285 were predicted based on annotation obtained from UniProt [28] and using BlastKOALA [29]. Approximately 54.8% and 57.6% of the 3888 *P. thermoglucosidasius* DSM proteins were assigned to GO terms or KEGG categories, respectively. 

Evaluation of the up-regulated transcripts at 20 h (relative to 8 h) post-inoculation, revealed significant (FDR < 0.05) enrichment of genes of annotated with 14 GO terms including four biological processes (BP), seven cellular components (CC) and three molecular functions (MF) (Table S2). By contrast, the down-regulated transcripts are significantly (FDR < 0.05) predominated by GOs linked to 91 BP, 3 CC and 1 MF. Evaluation of the enriched functions showed that among the BPs, GOs of anion transport (GO:0006820), developmental processes (GO:0032502) and sporulation (GO:0043934) led the up-regulated terms while drug metabolic process (GO:0017144) and locomotion (GO:0040011) were among the top down-regulated functions. Additionally, the GOs nucleotide and ATP biosynthesis were also down-regulated at 20 h relative to 8 h. The differentially expressed transcripts were also evaluated for KEGG pathway enrichment. At 20 h relative to 8 h, differentially expressed transcripts were significantly associated with between 14 (*q* < 0.05) and 18 (*p* < 0.05) KEGG pathways (Table 1). Genes linked to amino acid metabolism (valine, leucine and isoleucine degradation), carbohydrate metabolism (citrate cycle, glycolysis / gluconeogenesis, pyruvate, propanoate and butanoate metabolism), cell motility (flagellar assembly), energy metabolism (oxidative phosphorylation), biosynthesis of antibiotics and secondary metabolites, metabolism of cofactors and vitamins and translation (ribosomes) were down-regulated while only one category, membrane transport (ABC transporters), was significantly (*q* < 0.05) up-regulated. In addition to various roles in wide range of cellular processes, including transport of molecules [30], ABC transporters overexpression has been observed in *Mycobacterium smegmatis* grown under oxygen starvation [31].

Considering the differentially expressed genes at 27 h relative to 20 h, the GO enrichment analysis showed 10 GOs including 3 BPs, 5 CCs and 2 MFs were significantly (FDR < 0.05) up-regulated, with GOs of transport (GO:0006810), spore germination (GO:0009847) and developmental process (GO:0032502) being the top up-regulated processes (Table S2). Conversely, 44 GOs were significantly (FDR < 0.05) depleted, including 28 BPs and 8 CCs and MFs each. Differentially expressed transcripts at 27 h (relative to 20 h) enrich between nine (*q* < 0.05) and 18 (*p* < 0.05) KEGG pathways (Table 1). These include 13 of the pathways that have been down-regulated at 20 h, including the TCA cycle, ABC transport, biosynthesis of amino acids, antibiotics, secondary metabolites; porphyrin and chlorophyll metabolism and ribosome synthesis. Additionally, transcripts associated with fatty acid metabolism, carbohydrate (glyoxylate and dicarboxylate metabolism) and energy (methane metabolism) metabolism, folding, sorting and degradation (RNA degradation) as well as translation (aminoacyl-tRNA biosynthesis) were also down-regulated. Based on the KEGG pathway enrichment analysis, however, no specific function was up-regulated at this point. 

At the final stages of the experiment (44 vs.. 27 h), with cells undergoing the WGS reaction, 34 and one GOs were significantly (FDR < 0.05) enriched among the up- and down-regulated transcripts, respectively (Table S2). The most up-regulated function appeared to be translation (GO:0006412), while the alcohol catabolic process (GO:0046164) was down-regulated. Only one KEGG pathway, translation (ribosomes), was significantly (*q* < 0.05) up-regulated (Table 1).

Overall, the differential expression analysis provides some insight into the physiological adaptation of *P. thermoglucosidasius* DSM 6285 in response to changing gas atmospheres and shifts in metabolism. The observed up-regulation of sporulation transcripts (Table S2) and down-regulation of flagellar assembly (Table 1) at 20 h suggests a degree of metabolic shut-down in response to energy limitation and build-up of acid in the medium. At the 8 h time point, DSM 6285 attained the maximum OD600 of 0.37 while the medium pH dropped from 6.69 to 6.43 (Figure 1a), indicating the accumulation of acetic acid under aerobic conditions. This phenomenon is characteristic of the ‘overflow mechanism’ observed when bacteria are grown with excess carbon source as a strategy to fast track acetyl-coA metabolism and maximise ATP synthesis when the key enzymes of the TCA cycle are inhibited [32,33]. Aerobic acetate production has been reported in *P. thermoglucosidasius* M10EXG [34]. Although only 0.75 g L^−1^ of glucose was used in the current experiment, the presence of carbon monoxide (CO), which is known to inhibit aerobic respiration via the inactivation of enzymes of the TCA cycle and the respiratory chain [35,36], may have resulted in a backlog of glucose which is channelled to acetate.

Inhibition of the respiratory chains will also result in the excess of reducing equivalents [35,36]. For instance, *Mycobacterium tuberculosis* H37Rv was demonstrated to maintain the balance of redox intermediaries by utilizing oxygen and, in its absence, by either decreasing the reduction of cofactors like FAD, NAD and NADP or via using pathways that convert cofactors to their oxidized forms [37].

**Figure 3:**
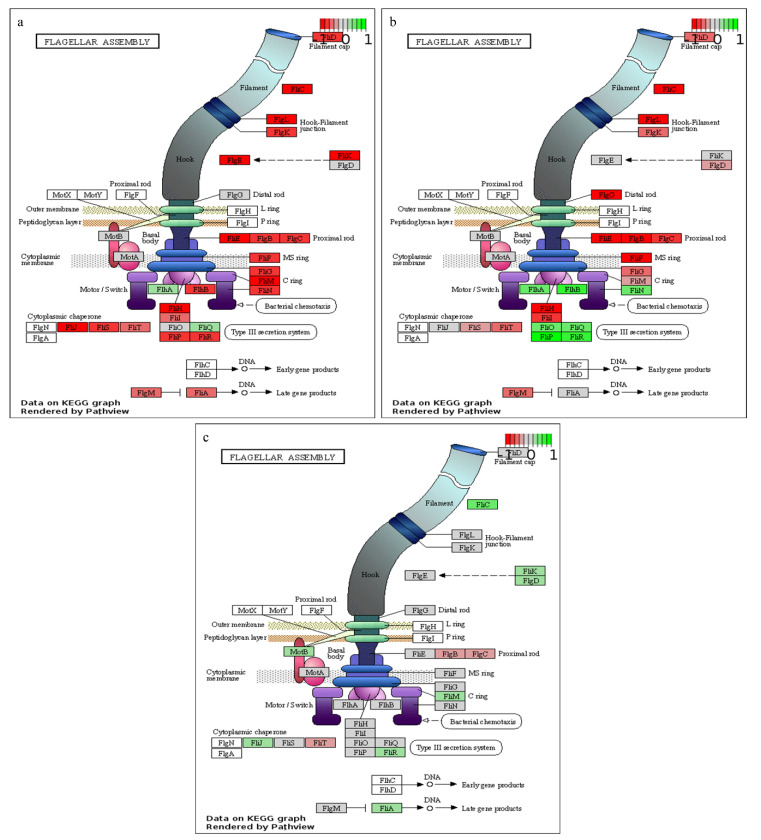
KEGG pathway enrichment of flagellar assembly. (**a**) 20 vs.. 8 h, (**b**) 27 vs.. 20 h and (**c**) 44 vs.. 27 h post-inoculation. Green, grey and red colours represent up-regulated, non-differentially expressed and down-regulated transcripts, respectively.

As observed from the growth curve and gas analysis (Figure 1a,b), *P. thermoglucosidasius* DSM 6285 renewed the growth between 27 and 44 h under WGS. This is supported by the observed induction of cellular protein metabolic process, ribosome, and translational activities (Table S2). Interestingly, there was gradual restoration of flagellar function at 27 h (Figure 3), as indicated by the overexpression of *fliO, fliQ, fliP* and *fliR* associated with the type III secretory system. Furthermore, transcripts of RNA polymerase sigma factor FliA and the switch mediator protein FliK were overexpressed at 44 h. FliA and its inhibitor, FlgM have been shown to play a central role in regulating the assembly of flagellum in bacteria [38].

### 2.3. Temporal Dynamics of P. thermoglucosidasius DSM 6285 Transcriptome between Aerobic Metabolism to Water Gas SHIFT reaction 

**Figure 4:**
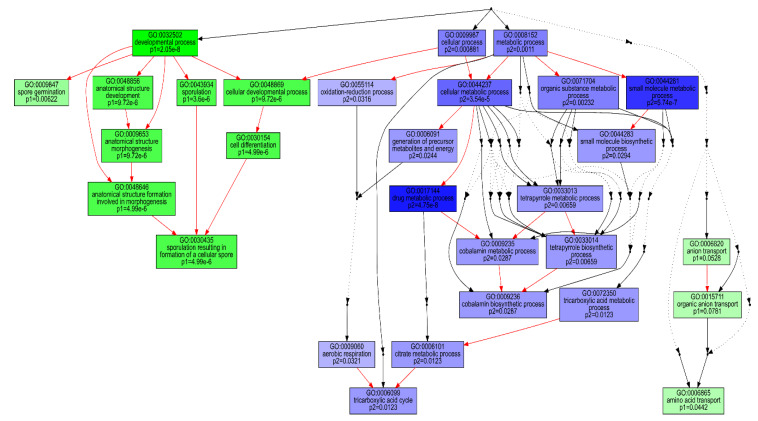
Comparison of GO-enriched function among two predominant dp_gp_cluster trajectory sets. P1 (green boxes) comprises the pooled transcripts of clusters 8, 11, 13, 18 and 19 which are up-regulated at 44 relative to 8 h and P2 (blue boxes) includes transcripts of clusters 1, 3, 4, 9, 16 and 22, generally down-regulated at 44 relative to 8 h. The hierarchical relationships between different GO terms are represented by arrows. Red, black solid and black dashed arrows represent relationships, between two enriched GO terms, between enriched and unenriched terms and between two unenriched GO terms, respectively.

Clustering of the 3055 normalised transcript counts showing differential expression in at least two consecutive condition sets revealed that the transcripts were grouped into 26 clusters with varying trajectories over the four time points (Figure S2). Of these, 11 clusters showing two distinct patterns of expression comprised over 75% of the analysed transcripts. Transcripts in first set of clusters (8, 11, 13, 18 and 19), comprising 1319 transcripts, were generally down-regulated at 8 h and up-regulated at 44 h, while the second set of 1031 transcripts (1, 3, 4, 9, 16 and 22) showed overexpression at 8 h and down-regulation at 44 h. GO enrichment analysis revealed that the first cluster set was dominated by transcripts associated with amino acid transport, cellular developmental and sporulation, while the second set was significantly enriched with transcripts of metabolic processes, including oxidation reduction, the tricarboxylic acid cycle and tetrapyrrole metabolism (Figure 4). KEGG enrichment analysis for these sets of clusters also revealed that only the ABC transporters were significantly (*q* < 0.05) enriched for the first set (Table 2). By contrast, significant (*q* < 0.05) enrichment of eleven pathways, biosynthesis of antibiotics and secondary metabolites, carbon metabolism and oxidative phosphorylation was observed for the second set of clusters.

**Figure 5:**
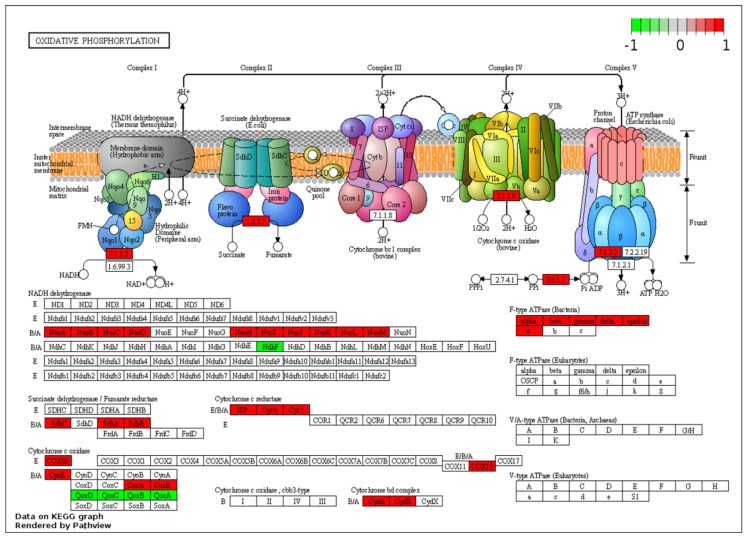
KEGG pathway enrichment of oxidative phosphorylation for two predominant dp_gp_cluster trajectory sets. Enzymes highlighted in green are linked to the pooled transcripts of clusters 8, 11, 13, 18 and 19 which are up-regulated at 44 relative to 8 h and those in red are linked to transcripts of clusters 1, 3, 4, 9, 16 and 22, generally down-regulated at 44 relative to 8 h post-inoculation.

While the clusters highlight the overall trend in terms of a shift in metabolism from aerobic processes to the anaerobic WGS-driven phase, it masks some of the nuances. For example, the significant down-regulation of some the pathways coincides with the up-regulation of specific transcripts for pathways that may be of interest for the metabolism of the organism during the WGS reaction. A specific example is the oxidative phosphorylation pathways, where transcripts for genes across the respiratory chain are generally down-regulated at 44 h post-inoculation relative to the aerobic phase (8 h). These include transcripts of genes involved in the synthesis of the NADH-quinone oxidoreductase (*nuoB, nuoC, nuoD, nuoJ, nuoK, nuoN* and *ndhF*), cytochrome *bc1* complex (*cytb, cyt1* and *isp*), cytochrome c oxidase *caa3* (*ctaA* and *ctaB*) and the terminal cytochrome *bd* oxidase (*cydA*). Together, these changes suggest a shift from aerobic respiration to anaerobic fermentation (Figure 5). 

However, two transcripts, *atpD* and *atpC*, coding for the β and ε subunits of the F_1_F_0_-type ATP synthetase are significantly up-regulated at the 44h timepoint relative to the aerobic (8h) timepoint. A combined transcriptome and mutagenesis study of *Listeria monocytogenes* also showed up-regulation of these genes when this organism was grown under anaerobic conditions [39]. It has been postulated that, under anaerobic conditions, F_1_F_0_-ATPase functions in reverse to export protons at the expense of ATP. Proton export via the F_1_F_0_-ATPase may regulate the intracellular pH [39,40] in response to pH effects resulting from aerobic acetate accumulation. The DSM 6285 transcriptome profile also showed an up-regulation of acetate kinase (AckA) transcripts at time point 20 relative to 8 h and those of acetyl-CoA synthetase (ACS) at 27 relative to 20 h, suggesting a switch to acetate assimilation in response to acid accumulation. In *E. coli*, phosphotransacetylase Pta and the acetate kinase AckA have been shown to reversibly convert acetate to acetyl CoA [32,41]. This is also consistent with the observed up-regulation of the transcripts *qoxA, qoxC* and *qoxA,* which are upregulated in *L. monocytogenes* in response to acid stress [42]. 

### 2.4. Transcriptome Pattern of P. thermoglucosidasius DSM 6285 CODH and Hydrogenases

**Figure 6:**
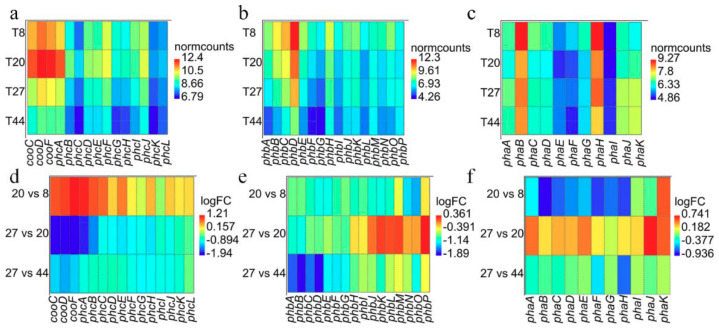
Pattern of expression among transcripts for the CODH and H_2_-evolving hydrogenase Phc (**a**,**d**) and the uptake hydrogenases Pha (**b**,**e**) and Phb (**c**,**f**). (**a**–**c**) Heat maps of the normalised transcripts counts (‘normcounts’) across the four time points. (**d**–**f**) Heat maps showing pattern of differential expression (logFC) for the comparisons of time points 20 vs.. 8, 27 vs.. 20 and 44 vs.. 27 h.

Evaluation of the gene expression profiles of the genes coding for the carbon monoxide dehydrogenase (CODH) and hydrogen evolving hydrogenase (Phc) [25] across the different points revealed that transcripts associated with CODH and Phc were more abundant at 8 and 20 h (aerobic phase) compared to 27 and 44 h (anaerobic and WGS phase; Figure 6a). Furthermore, the transcripts are generally up-regulated at time point 20 relative to 8 h and down-regulated at 27 relative to 20 h and at 44 relative to 27 h (Figure 6d). However, differential expression analysis revealed that only transcripts of *cooF* (coding for the CODH iron-sulphur protein) and *phcABCE* (coding for the Ni-Fe hydrogenase 4 B, C, E and D subunits, respectively) were significantly (FDR < 0.05) up-regulated at 20 h. By contrast, all transcripts except *phcIKL* (coding for the hydrogenase maturation protein, maturation nickel metallochaperone and accessory protein, respectively) were significantly (FDR < 0.05) down-regulated at 27 h. Cluster analysis showed that the CODH and Phc transcripts followed similar trajectories as depicted in clusters 9 and 7 (Figure S3). The trajectories of *cooC* and *phcDFGHJ* suggest that these genes were up-regulated at both timepoint 8 and 20 h, and down-regulated at 44 h. By contrast, *cooSF* and *phcABCE* show rapid up-regulation at timepoint 20 relative to 8 h and subsequent down-regulation at 44 h. The initial overexpression of the latter group of transcripts, however, coincides with the depletion of O_2_ at 20 h, suggesting that these genes may constitute an O_2_ sensing mechanism; ie., anaerobic CODH and Ni-Fe group 4a hydrogenase are O_2_-sensitive [43,44,45]. It may be significant that transcripts of two putative CO-sensing transcriptional factors (Crp/Fnr; WP_090948317.1 and WP_064552491.1) were up-regulated (cluster 7; Figure S3) only at 44 h relative to the three other time points. The two Crp/Fnr proteins share similar protein sizes (230-236 amino acids in length) and domain architecture (PR018490, IPR000595 and IPR012318) as the CooA protein (223 aa) of *C. amalonaticus* Y19 [46]. These genes occur ~0.3 Mb upstream the CODH-H_2_-evolving hydrogenase locus in the DSM 6285 genome. 

In addition to the CODH-Phc complex, two loci coding for predicted uptake hydrogenases, Pha and Phb, have been previously predicted on the DSM 6285 genome [25]. These share orthology to the unidirectional uptake Ni-Fe group 1d hydrogenases and Ni-Fe group 2a hydrogenases, respectively [24,25]. Both uptake hydrogenases are found in aerobic and facultative anaerobic bacterial taxa, are O_2_ tolerant and play a role in recycling of H_2_ for use in aerobic and hydrogenotrophic respiration [43,44,47]. 

Transcripts of the Phb genes showed similar expression patterns as observed for the Phc genes, with general up-regulation at time point 8 h and down-regulation at time point 44 h (Figure 6b). However, differential expression analysis (Figure 6e) revealed significant (FDR < 0.05) down-regulation of all transcripts at time point 20 relative to 8 h. Only transcripts of *phbABCEFG* showed significant down-regulation at 27 compared to 20 h post-inoculation. Similarly, all transcripts were down-regulated at time point 44 relative to 27 h. All transcripts except *phbJKLM* showed similar trajectories depicting the pattern of expression observed above. Unlike most of the transcripts, *phbJKLM* appear to be constitutively expressed at 20 and 27 h and down-regulated like others Pha transcript at 44 h (Figure S3). 

Pha transcripts showed the most diverse expression pattern (Figure 6c). While *phaA* showed the highest expression at 27 h, *phaBCDEFGH* had maximum expression at 8 h. By contrast, the maximum expression of *phaIJK* was observed at 27 and 44 h. Comparison of transcript profiles at the different time points revealed, however, that *phaBCDFGH* expression was significantly (FDR < 0.05) down-regulated at 20 relative to 8 h while only *phaK* was significantly (FDR < 0.05) up-regulated at this time (Figure 6f). Comparing time points 27 and 20 h, only transcripts of two genes *phaJK* showed significant (FDR < 0.05) up-regulation. No significant differentially gene expression was observed at time point 44 relative to 27 h. The overall expression profiles for Pha revealed that these transcripts follow four distinct trajectories (Figure S3). 

The general trend suggests that the uptake hydrogenases Pha and Phb are active during aerobic phases of *P. thermoglucosidasius* growth as previously predicted [24]. However, the *phaJK* genes, which code for hydrogenase expression/formation proteins (HypD/HypE) are the only uptake hydrogenase transcripts with increased transcriptional activity under anaerobic conditions. This is perhaps surprising since HypD and HypE, which are involved in Ni incorporation and purine derivative binding, respectively, are part of the machinery that generates the matured hydrogenase [48]. 

## 3. Discussion

RNA-seq based transcriptome profiling of the facultative anaerobe *P. thermoglucosidasius* DSM 6285 growing under an initial gas atmosphere of 50% air and 50% CO revealed a metabolic flexibility reflective of its ability to switch between aerobic growth, anaerobic survival, and anaerobic growth to recovery via the WGS reaction. Annotation of differentially expressed transcripts using KEGG and GOs revealed the suppression of aerobic metabolism during O_2_ depletion [49], as indicated by downregulation of cytochrome oxidases and ATPase transcripts. Strikingly, transcripts of cytochrome aa3-600 menaquinol oxidases remained induced throughout the period of cultivation. Menaquinol oxidases serve as proton-pumping O_2_ reductases that oxidize membrane-bound quinols directly without the need for electrons from cytochrome c [50]. Although part of aerobic respiratory chains, their evolution has been predicted to predate atmospheric O_2_ [51] and it is therefore perhaps not surprising that they are induced under low O_2_ or anaerobic conditions in *P. thermoglucosidasius*. 

Previous work in *B. subtilis* revealed that at least one quinol oxidase is essential for the formation of spores [52,53]. Indeed, the up-regulation of aa3-600 coincides with the up-regulation of predicted sporulation genes as *P. thermoglucosidasius* DSM 6285 transitions from aerobic growth to the anaerobic phase where initiation of spore formation may serve as a survival strategy under energy limitation. This is further supported by the observed down-regulation of motility. The change from motile to non-motile forms is typically observed in the transition between exponential and stationary phase and often the two phases coexist [54,55]. 

The up-regulation of transcripts of the anaerobic CODH and H_2_-evolving Phc seems to coincide with O_2_ depletion and the locus does not appear to be under the control of the putative CO-sensing transcriptional factor (Crp/Fnr) as reported for other carboxydotrophs [56]. Sensitivity of the anaerobic CODH to oxygen has been observed at both protein (enzymic activity) and genetic levels [57,58]. However, the precise regulation of the CODH genes in *P. thermoglucosidasius* DSM 6285 remains unclear as the current fermentation strategy, which was optimised for early hydrogen production, also incorporated CO from the inception. Further investigations should focus on elucidating the regulation of CODH loci in *P. thermoglucosidasius* DSM 6285.

The commencement of the biological WGS reaction after ~27 h, as indicated by the consumption of CO and evolution of H_2_, coincided with the resumption of several metabolic activities. Both GO and KEGG enrichment analyses showed that ribosome biosynthesis was more active at 44 compared to 27 h. Up-regulation of ribosome biosynthesis has been reported during recovery of *Nitrosomonas europaea* impaired by CeO_2_ nanoparticles [59]. Overexpression of ribosome biosynthesis has also been suggested as a means by which microorganisms compensate for low temperature-induced reduction in the rate of protein biosynthesis [60,61,62]. Although the current experiment did not directly quantify the amount of energy obtained by the organism via the WGS reaction, the up-regulation of genes coding for key central metabolic enzymes—e.g., ATP-dependent 6-phosphofructokinase and pyruvate, as well as the observable increase in biomass (Figure 1)—suggest that the WGS provides sufficient energy to support growth.

## 4. Materials and Methods 

### 4.1. Microorganism and Media

*P. thermoglucosidasius* DSM 6285 was obtained from the Deutsche Sammlung von Mikroorganismen und Zellkulturen (DSMZ, Braunschweig, Germany) and stored at −80 °C in glycerol. 

For cultivation of the pre-cultures, mLB (modified Luria-Bertani) medium was used as previously described [24]. The experiment was performed in a modified ammonium sulphate medium (ASM) [63], comprising 8.7 mM citric acid, 20.2 mM MgSO_4_, 10 mM K_2_SO_4_, 22.6 mM NaH_2_PO_4_, 0.8 mM CaCl_2_, 25 mM (NH_4_)_2_SO_4_, 4.16 mM glucose and 1 mL each of the filter-sterilized trace elements: 6 mM H_2_SO_4_, 0.1 mM CuSO_4_, 0.2 mM CoSO_4_, 0.5 mM ZnSO_4_, 2.29 mM FeSO_4_, 0.3 mM NiSO_4_, 0.9 mM MnSO_4_ and 0.1 mM H_3_BO_3_. The pH was adjusted to 6.8 with 4 M NaOH.

### 4.2. Experimental Set up and Analytical Methods

A two-step pre-culture system was used in the study. All pre-culture cultivation was carried out in 20 mL mLB medium. The first pre-culture was inoculated with 20 µL of a glycerol stock and cultured aerobically for 24 h at 55 °C, 120 rpm (Infors Thermotron, Infors AG, Bottmingen, Switzerland). Thereafter, a second pre-culture was inoculated to an OD_600_ of 0.1 and cultivated aerobically for 12 h (60 °C, 120 rpm). The experimental set-up (49 mL ASM medium) was inoculated with 1 mL of the second pre-cultures in 250-mL stoppered serum bottles with an initial gas atmosphere of 50% air and 50% CO. Samples for RNA-Seq analysis were taken in the aerobic phase (8 h), the anaerobic lag phase (20 h), the anaerobic phase prior to H_2_ production (27 h) and the H_2_-production phase (44 h). The experiment was conducted with two biological replicates, each comprised of six identical bottles. To obtain adequate amounts of RNA for sequencing, 4 mL of culture was collected from each bottle and pooled. Pressure was measured before and after every sample step using a manometer (GDH 14 AN, Greisinger electronic, Regenstauf, Germany). Absorbance (OD_600_) was measured as an indication of bacterial growth using an Ultrospec 1100 pro spectrophotometer (Amersham Biosciences, Picataway, NJ, USA) at each sample point. pH was monitored with a pH meter (Profilab pH 597, Xylem Analytics Germany Sales GmbH and Co. KG, WTW, Oberbayern, Germany). To determine the gas composition in the bottle, 4 mL gas samples were taken and injected to a 300 Micro GC gas analyzer (Inficon, Bad Ragaz, Switzerland). The calculation of the gas composition was carried out as previously described [24]. 

### 4.3. RNA Isolation, Library Preparation, and RNA-Seq

Total RNA extraction, rRNA depletion and sequencing were conducted by Microsynth AG (Balgach, Switzerland). Stranded, ribodepleted RNA libraries were prepared using MICROBExpress (Thermo Fisher, Balgach, Switzerland) according to the manufacturer’s instructions. Sequencing was performed using Illumina NextSeq, v2.5, 1x75bp chemistry. The reads were then demultiplexed and trimmed of Illumina adaptor residuals. All sequencing data obtained from this study have been deposited in the European Nucleotide Archive (ENA) under study accession number PRJEB36750.

### 4.4. RNA-Seq Data Analysis

The RNA-Seq reads were initially analysed using SPARTA [64] with default settings. Briefly, the reads were trimmed and analysed for quality using Trimmomatic [65] and FastQC [66], respectively. High-quality reads were mapped against the draft genome sequence of *P. thermoglucosidasius* DSM 6285 using Bowtie [67]. Transcript feature abundance was subsequently determined with HTSeq [68]. To determine overexpressed transcripts between time points, differential gene expression analysis was performed using edgeR [69]. The normalised counts obtained from edgeR [69] were used to profile the time-dependent expression of transcripts using DP_GP_cluster [70]. The draft genome sequence of *P. thermoglucosidasius* DSM 6285 was annotated using gene ontology (GO) and KEGG orthologues obtained from UniProt [28] and using BlastKOALA [29]. GOEAST [71] and Pathview Web [72] were used to predict the functions of overexpressed transcripts. MDS plots and heat maps were generated using edgeR [69] and PAST [73], respectively.

## 5. Conclusions

We analysed the genetic response of *P. thermoglucosidasius* DSM 6285 transitioning from aerobic respiration to anaerobic growth and supported by CO metabolism and the WGS reaction. The transcriptome data revealed no obvious impact of 50% CO in the atmosphere on the initial aerobic metabolic phase in the presence of O_2_ with *P. thermoglucosidasius* DSM 6285 only switching to CO metabolism after O_2_ depletion, possibly due to the O_2_-dependent inactivation of the WGS enzymes (CODH and hydrogenase) by O_2_. Furthermore, a shift from a motile to non-motile physiology, a reduction in metabolic activity and the activation of stress response and sporulation pathways are all observed once O_2_ is depleted, where *P. thermoglucosidasius* adapts to circumvent energy limitations through the WGS reaction. Overall, the data revealed a clear partitioning of transcripts which reflects a metabolic shift to cope with energy limitation and subsequent resumption of growth driven by the WGS reaction.

## Figures and Tables

**Table 1:** KEGG pathway enrichment of differentially expressed genes at 20 vs.. 8 h (P1), 27 vs.. 20 h (P2) and 44 vs.. 27 h (P3). Positive and negative statistical mean values indicate up- and down-regulated functions, respectively.

Set	Kegg Pathway	Definition	Stat. Mean	Set. Size	*q*
P1	ko01100	Metabolic pathways	−4.35	273	6.31 × 10^−4^
	ko00020	Citrate cycle (TCA cycle)	−5.11	19	7.18 × 10^−4^
	ko01130	Biosynthesis of antibiotics	−4.09	90	7.18 × 10^−4^
	ko02040	Flagellar assembly	−5.24	20	7.18 × 10^−4^
	ko00860	Porphyrin and chlorophyll metabolism	−4.36	20	1.37 × 10^−3^
	ko01110	Biosynthesis of secondary metabolites	−3.37	118	5.51 × 10^−3^
	ko01230	Biosynthesis of amino acids	−3.40	50	5.51 × 10^−3^
	ko00010	Glycolysis / Gluconeogenesis	−3.37	28	6.31 × 10^−3^
	ko00190	Oxidative phosphorylation	−3.37	28	6.31 × 10^−3^
	ko03010	Ribosome	−3.38	14	1.16 × 10^−2^
	ko01200	Carbon metabolism	−2.82	56	2.01 × 10^−2^
	ko00620	Pyruvate metabolism	−2.57	25	3.71 × 10^−2^
	ko00640	Propanoate metabolism	−2.63	17	3.71 × 10^−2^
	ko00650	Butanoate metabolism	−2.46	16	5.02 × 10^−2^
	ko01210	2-Oxocarboxylic acid metabolism	−2.58	10	5.02 × 10^−2^
	ko00720	Carbon fixation pathways in prokaryotes	−2.37	21	5.24 × 10^−2^
	ko01120	Microbial metabolism in diverse environments	−2.27	110	5.26 × 10^−2^
	ko00280	Valine, leucine and isoleucine degradation	−1.84	10	1.68 × 10−01
	ko02010	ABC transporters	2.71	67	2.51 × 10^−2^
P2	ko03010	Ribosome	−5.38	49	9.20 × 10−05
	ko01110	Biosynthesis of secondary metabolites	−4.59	133	1.49 × 10^−4^
	ko01100	Metabolic pathways	−4.10	282	5.93 × 10^−4^
	ko01230	Biosynthesis of amino acids	−4.27	53	5.93 × 10^−4^
	ko01130	Biosynthesis of antibiotics	−3.53	97	4.42 × 10^−3^
	ko01200	Carbon metabolism	−3.52	62	4.54 × 10^−3^
	ko00860	Porphyrin and chlorophyll metabolism	−3.60	22	1.00 × 10^−2^
	ko00020	Citrate cycle (TCA cycle)	−3.35	19	1.66 × 10^−2^
	ko00190	Oxidative phosphorylation	−2.37	32	8.68 × 10^−2^
	ko00620	Pyruvate metabolism	−2.33	27	8.68 × 10^−2^
	ko00720	Carbon fixation pathways in prokaryotes	−2.33	22	8.68 × 10^−2^
	ko01210	2-Oxocarboxylic acid metabolism	−2.55	11	8.68 × 10^−2^
	ko01212	Fatty acid metabolism	−2.55	11	8.68 × 10^−2^
	ko00630	Glyoxylate and dicarboxylate metabolism	−2.22	21	9.25 × 10^−2^
	ko00970	Aminoacyl-tRNA biosynthesis	−2.43	10	9.38 × 10^−2^
	ko03018	RNA degradation	−2.43	10	9.38 × 10^−2^
	ko00680	Methane metabolism	−1.91	13	1.68 × 10^−1^
	ko02010	ABC transporters	3.15	64	1.07 × 10^−2^
P3	ko03010	Ribosome	5.71	29	2.40 × 10^−5^

**Table 2:** KEGG pathway enrichment among two predominant dp_gp_cluster trajectory sets. Cluster set 1 comprises the pooled transcripts of clusters 8, 11, 13, 18 and 19 which are up-regulated at 44 relative to 8 h and Cluster set 2 includes transcripts of clusters 1, 3, 4, 9, 16 and 22, generally down-regulated at 44 relative to 8 h.

KEGG Pathway	Definition	Cluster Set	Set. Size	*p*	*q*
ko02010	ABC transporters	1	67	1.35 × 10^−3^	7.72 × 10^−3^
ko00020	Citrate cycle (TCA cycle)	2	22	3.89 × 10^−4^	2.59 × 10^−3^
ko00190	Oxidative phosphorylation	2	34	5.99 × 10^−3^	2.42 × 10^−2^
ko00620	Pyruvate metabolism	2	24	3.58 × 10^−3^	1.79 × 10^−2^
ko00640	Propanoate metabolism	2	22	6.66 × 10^−3^	2.42 × 10^−2^
ko00860	Porphyrin and chlorophyll metabolism	2	25	1.51 × 10^−4^	1.51 × 10^−3^
ko01100	Metabolic pathways	2	304	1.59 × 10^−5^	5.25 × 10^−4^
ko01110	Biosynthesis of secondary metabolites	2	134	2.62 × 10^−5^	5.25 × 10^−4^
ko01130	Biosynthesis of antibiotics	2	100	7.54 × 10^−5^	1.01 × 10^−3^
ko01200	Carbon metabolism	2	52	3.43 × 10^−4^	2.59 × 10^−3^
ko03010	Ribosome	2	12	9.88 × 10^−3^	3.29 × 10^−2^

## Supplementary Materials

The following are available online at https://www.mdpi.com/1422-0067/21/11/3870/s1, Figure S1: MDS plot showing pattern of transcripts expression in four RNA-seq samples of *P. thermoglucosidasius* DSM 6285 cultivated in two biological replicates (indicated by ‘_replicate number’) in stoppered serum bottles with an initial gas atmosphere composition of 50% CO and 50% air over four time points. Figure S2: DP_GP_cluster analysis of differentially expressed transcripts of *P. thermoglucosidasius* DSM 6285 cultivated in stoppered serum bottles with an initial gas atmosphere composition of 50% CO and 50% air and samples over four time points. Figure S 3. Trajectories of transcripts in the carbon monoxide dehydrogenase (CODH), uptake hydrogenase (Pha and Phb) and H_2_-evolving hydrogenase (Phc) loci. Table S1: Overview of RNA-seq read metrics of *P. thermoglucosidasius* DSM 6285. Culture were cultivated in two biological replicates in stoppered serum bottles with an initial gas atmosphere composition of 50% CO and 50% air over four time points. Table S2: Gene ontology (GO) enrichment of differentially Table S2: Gene ontology (GO) enrichment of differentially expressed genes of *P. thermoglucosidasius* DSM 6285 at 20 vs.. 8 h (P1), 27 vs.. 20 h (P2) and 44 vs.. 27 h (P3). The organism was grown with an initial gas atmosphere composition of 50% CO and 50% air genes at 20 vs.. 8 h (P1), 27 vs.. 20 h (P2) and 44 vs.. 27 h (P3).
