# Supplementary material for: Time-course Transcriptome of Parageobacillus thermoglucosidasius DSM 6285 Grown in the Presence of Carbon Monoxide and Air
Source: Int J Mol Sci. 2020 May 29;21(11):3870. doi: 10.3390/ijms21113870 (PMC7312162; doi:10.3390/ijms21113870)
Supplement: Supplementary file 1 [file ijms-21-03870-s001.zip › ijms-797849_proof-supplementary/Supplementary_File_1.docx]

ADDITIONAL FILE 1

**Table S1:** Overview of RNA-seq read metrics of *P****.*** *thermoglucosidasius* DSM 6285. Culture were cultivated in two biological replicates in stoppered serum bottles with an initial gas atmosphere composition of 50% CO and 50% air over four time points.

| **Sample** | **#Reads** | **#Bases** | **Mean Read Length** | **Mean Q** | **% trimmed and aligned** |
| --- | --- | --- | --- | --- | --- |
| T8_1 | 18,784,192 | 1,392,112,556 | 74 | 35 | 94.23 |
| T8_2 | 10,499,391 | 780,582,143 | 74 | 34 | 92.63 |
| T20_1 | 14,357,303 | 1,064,984,019 | 74 | 35 | 95.76 |
| T20_2 | 17,787,669 | 1,309,276,428 | 74 | 35 | 94.19 |
| T27_1 | 13,877,569 | 1,026,140,873 | 74 | 34 | 93.84 |
| T27_2 | 16,067,028 | 1,183,613,704 | 74 | 34 | 91.7 |
| T44_1 | 6,910,074 | 514,335,535 | 75 | 35 | 98.11 |
| T44_2 | 7,949,465 | 591,253,760 | 75 | 35 | 98.02 |


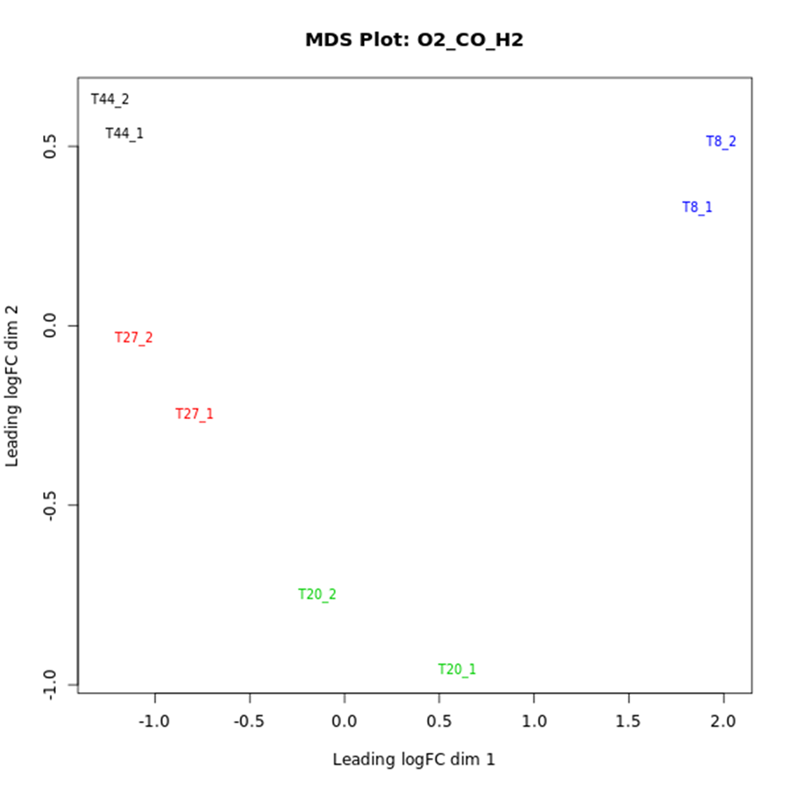


**Figure S1.** MDS plot showing pattern of transcripts expression in four RNA-seq samples of *P. thermoglucosidasius* DSM 6285 cultivated in two biological replicates (indicated by ‘_replicate number’) in stoppered serum bottles with an initial gas atmosphere composition of 50% CO and 50% air over four time points.


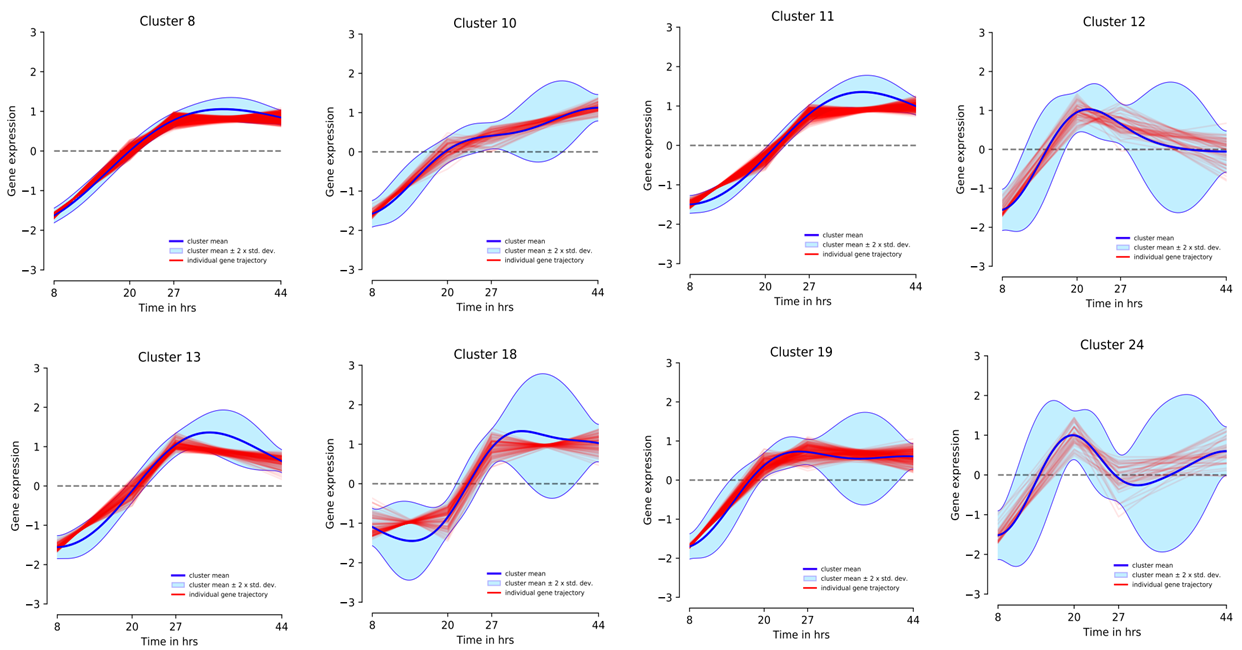


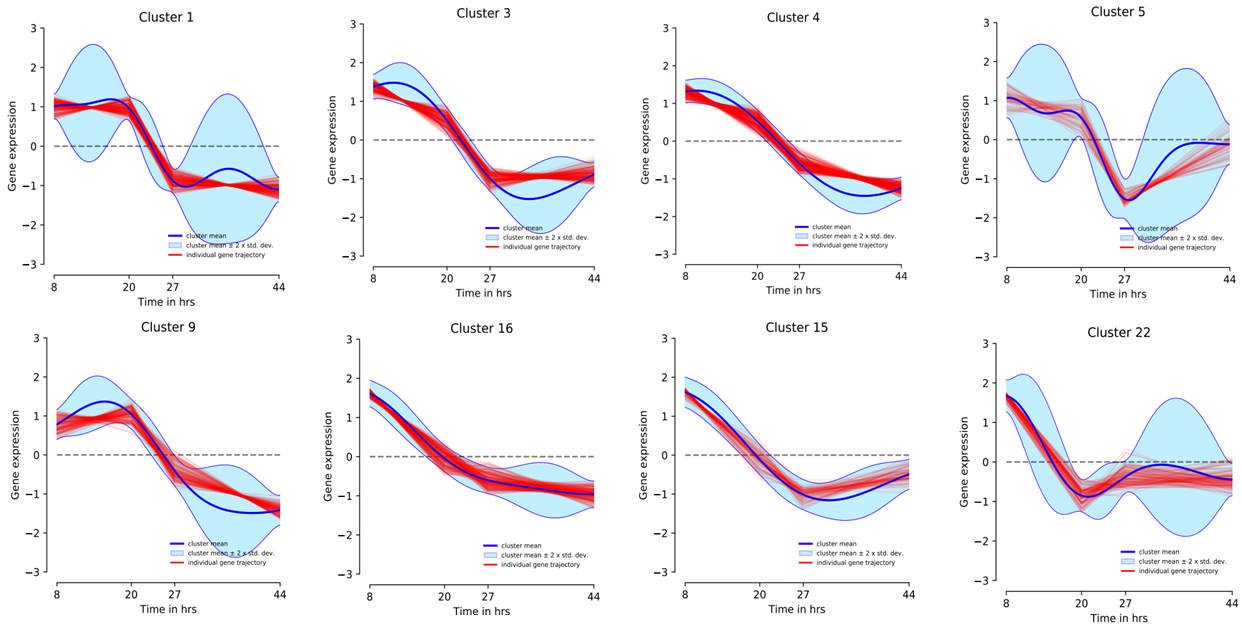


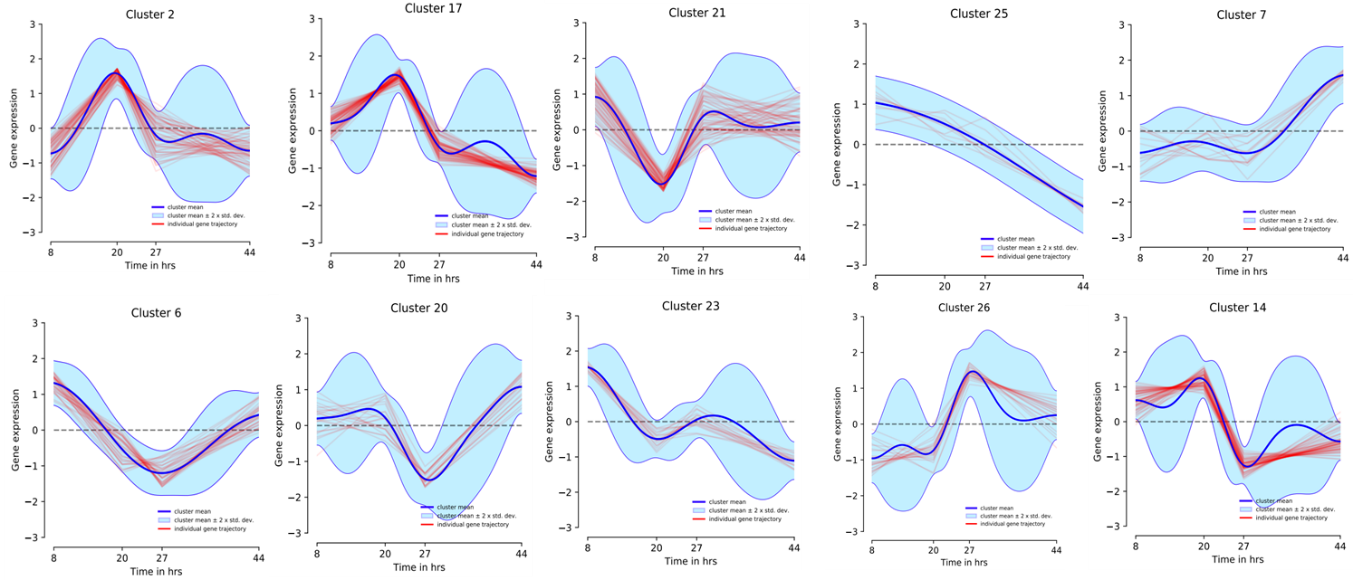


**Figure S2.** DP_GP_cluster analysis of differentially expressed transcripts of *P. thermoglucosidasius* DSM 6285 cultivated in stoppered serum bottles with an initial gas atmosphere composition of 50% CO and 50% air and samples over four time points.


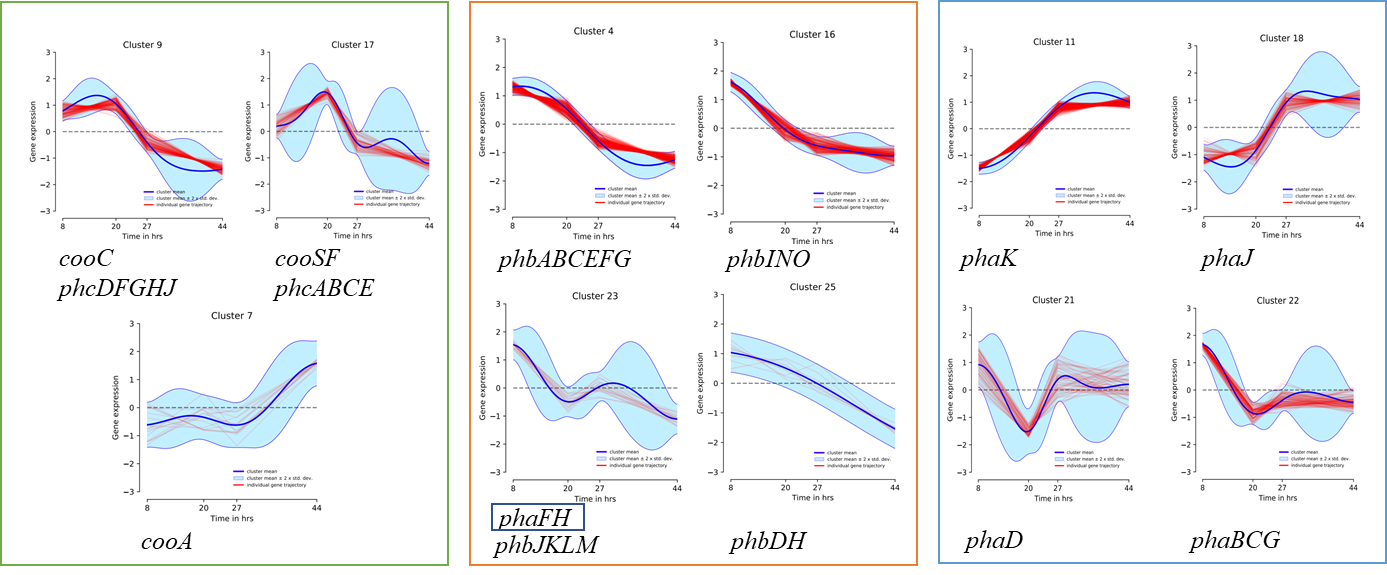


**Figure S3.** Trajectories of transcripts in the carbon monoxide dehydrogenase (CODH), uptake hydrogenase (Pha and Phb) and H_2_-evolving hydrogenase (Phc) loci.
